# Stable contacts of naïve CD4 T cells with migratory dendritic cells are ICAM-1-dependent but dispensable for proliferation in vivo

**DOI:** 10.1080/19336918.2019.1644857

**Published:** 2019-07-31

**Authors:** Stav Kozlovski, Ofir Atrakchi, Sara W Feigelson, Ziv Shulman, Ronen Alon

**Affiliations:** Department of Immunology, Weizmann Institute of Science, Rehovot, Israel

**Keywords:** Lymph nodes, dendritic cells, integrins, T cell activation, vaccination

## Abstract

It is unclear if naïve T cells require dendritic cell ICAMs to proliferate inside lymph nodes. To check if and when CD4 lymphocytes use ICAMs on migratory DCs, wild-type and ICAM-1 and 2 double knock out bone marrow-derived DCs pulsed with saturating levels of an OT-II transgene-specific ovalbumin-derived peptide were co-transferred into skin-draining lymph nodes. Intravital imaging of OT-II lymphocytes entering these lymph nodes revealed that ICAM-1 and −2 deficient migratory DCs formed fewer stable conjugates with OT-II lymphocytes but promoted normal T cell proliferation. DC ICAMs were also not required for unstable TCR-dependent lymphocyte arrests on antigen presenting migratory DCs. Thus, rare antigen-stimulated ICAM-stabilized T-DC conjugates are dispensable for CD4 lymphocyte proliferation inside lymph nodes.

## Introduction

Naïve T cells make fate decisions within hours after antigen exposure, resulting in activation, proliferation and either long-term memory or abortive effector responses, which correlate with T cell-DCs interaction kinetics [1–4]. The LFA-1 integrin on T cells and its two main ligands ICAM-1 and ICAM-2 expressed by DCs as well as by other antigen presenting cells (APCs) were suggested to modulate the strength, the kinetics, and hence the outcome of these interactions [5,6]. Specifically, the LFA-1-ICAM-1 axis was suggested to amplify TCR signals transmitted within T-DC contacts, collectively termed immune synapses [7,8]. However, the contribution of this molecular axis to T-DC communication was mostly investigated in vitro [9]. The specific in vivo contributions of LFA-1-ICAM interactions to naïve T cell proliferation are still disputed [10–13], due to the lack of adequate genetic models of APC-type specific ICAM knock out mice.

To address this issue, we have recently used chimeric mice expressing normal non-hematopoietic ICAM-1 and ICAM-2 reconstituted with immune cells deficient in both ICAMs [13]. In this work we found that combined ICAM-1 and ICAM-2 deficiency on resident lymph node DCs stimulated with anti CD40 and immunized with soluble antigen did not impair their ability to arrest naïve CD4 lymphocytes entering these lymph nodes from blood and did not abrogate their subsequent in vivo differentiation into Th1 and Tfh effectors [13]. Our conclusion was that functional T cell receptor (TCR)-triggered contacts between naïve CD4 T cells and resident antigen-presenting lymph node DCs do not require the presence of ICAM-1 or ICAM-2 on resident lymph node DCs [13]. This study suggested that the ability of naïve T cells to in situ activate their LFA-1 adhesiveness in response to potent TCR signals, a processed termed LFA-1 inside out activation [14], is much more restricted than previously thought [15,16]. The configuration of that study involved soluble antigen presented to naive OT-II CD4 T cells by essentially all DCs residing within the T zone of popliteal draining lymph nodes [13]. The failure to detect any contribution of the LFA-1-ICAM-1 and 2 axis to CD4 T cell proliferation and differentiation was attributed to individual naïve T cells engaging in highly frequent contacts with the numerous antigen presenting DCs they encountered, and reaching a TCR activation threshold by serial short-lived encounters with multiple DCs [3,17,18]. The study left open the possibility that ICAM-1 is potentially used by specialized subsets of migratory DCs which, once stimulated and loaded with high dose of cognate antigen in the periphery (e.g., skin), enter draining lymph nodes and present this antigen to highly motile CD4 T cell clones with cognate TCR. We reasoned that these singular T cells that scan the T zone of these lymph nodes would not be able to engage in multiple short-lived serial contacts with these rare migratory DCs as they do with the numerous antigen-loaded resident DCs. Thus, the ability of singular T cells to undergo activation and proliferation would depend on the generation of long-lived synapses with the rare antigen loaded migratory DCs they encounter inside the T zone. For consistency, we decided to address this question with the same transgenic OT-II CD4 T cells studied in our previous study. As a model for singular migratory DCs, we used bone marrow dendritic cells (BMDCs [18]) derived from either WT or ICAM-1 and ICAM-2 double knockout bone marrow. These DCs are extensively used in various types of vaccinations [19,20] and for dissecting DC-T cell synapses in various lymphoid organs [21–23].

Using a competitive transfer model and intravital microscopy of the T zone of these lymph nodes, we found that antigen-loaded LPS-stimulated BMDCs entered these zones through afferent lymphatics independently of ICAM-1 or ICAM-2 expression and arrested motile OT-II T cells in a TCR-dependent manner. A fraction of these TCR triggered OT-II T cells utilized BMDC ICAM-1 for generating stable conjugates with antigen-loaded DCs, but these ICAM-1- dependent T-DC synapses were not required for the subsequent proliferation of these CD4 T cells. These results suggest that naïve CD4 T cells can in situ activate their adhesiveness to ICAMs on migratory DCs in response to strong TCR signals, but this adhesion is not essential for their subsequent TCR stimulated proliferation.

## Results and discussion

**Figure 1.**
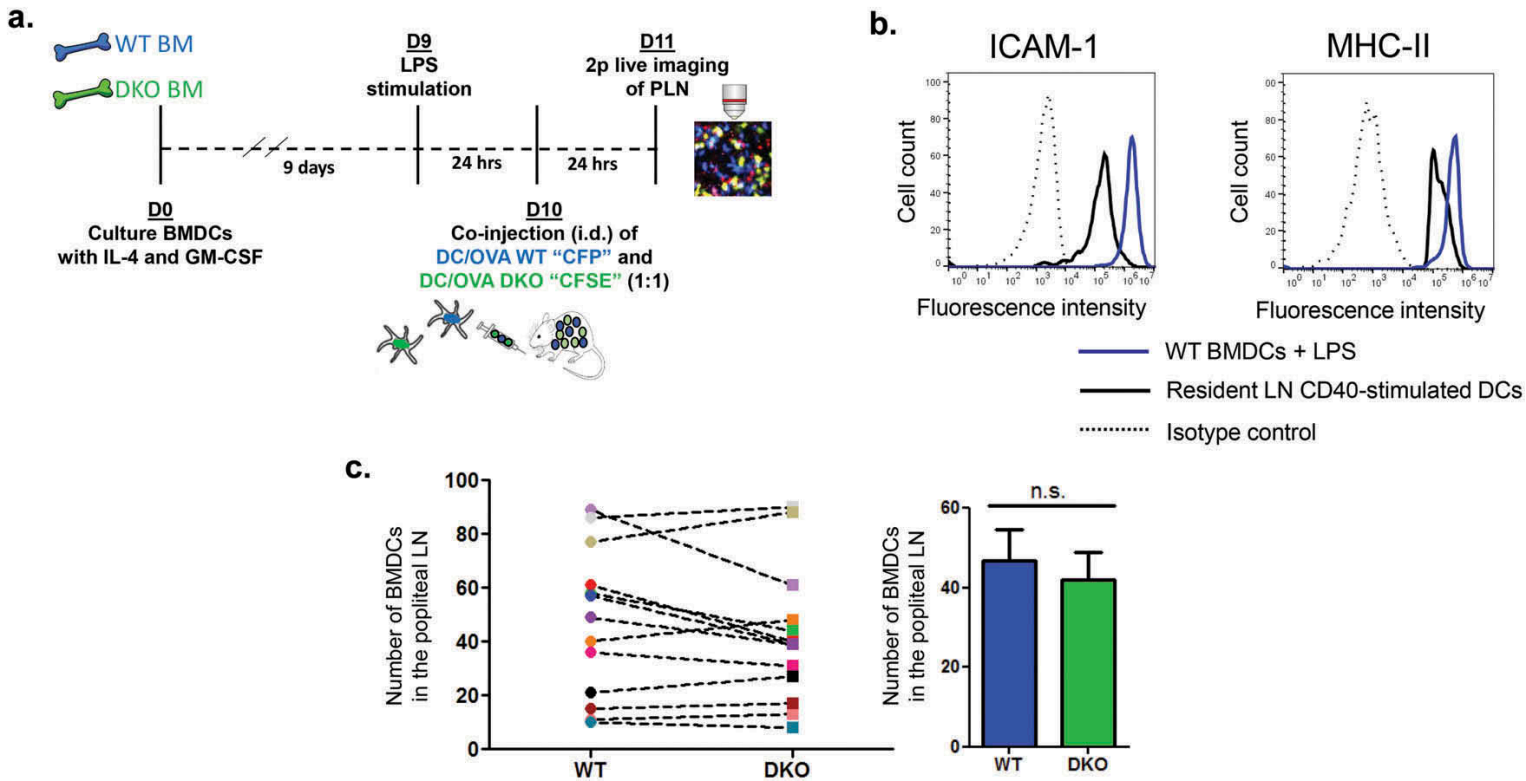
The experimental system for the generation and imaging of migratory BMDCs. (a) A scheme depicting the in vitro preparation of WT (CFP) and DKO (CFSE) BMDCs, LPS stimulation, intradermal (i.d.) co-injection, and 2 Pmicroscopy live imaging in popliteal lymph nodes of WT recipients. (b) ICAM-1 and MHC-II expression on BMDCs vs. resident lymph node DCs stimulated with anti-CD40. ICAM-2 was undetectable on BMDCs (not shown). (c) ICAM deficiency does not affect the entry of co-injected LPS stimulated OVA peptide loaded DCs into the T zone of popliteal lymph nodes, determined 24-h post intrafootpad transfer in multiple fields in this zone. Right panel depicts the mean of the values in multiple fields of view determined in eight mice.

In order to assess the role of DC ICAMs in naïve T cell activation by migratory DCs [20] primed in the skin and entering popliteal lymph nodes, we generated in vitro BMDC, from the BM of either WT or ICAM-1 and −2 double KO (herein ICAM-1/2 DKO) mice and stimulated these antigen presenting cells with LPS 24 h prior to injection (Figure 1(a)). These BMDCs (herein referred to as ICAM-1/2 DKO BMDCs) were found to express 8-fold higher ICAM-1 and 2-fold higher MHC-II than resident adjuvant stimulated lymph node DCs but lacked surface ICAM-2 expression (Figure 1(b), and not shown). Notably, ICAM-1 deficiency in these DCs did not affect their MHC-II, CD80, CD86 or CD40 expression (Suppl. Figure 1), indicating that ICAM-1 or −2 expression is not required for the expression of either of these key T cell activating and co-stimulatory molecules. Furthermore, when injected into the footpad, WT and ICAM DKO DCs accumulated at similar rates (Figure 1(c)) inside the T zone of popliteal lymph nodes (PLN), where DC encounter with naïve T cells take place.

**Figure 2.**
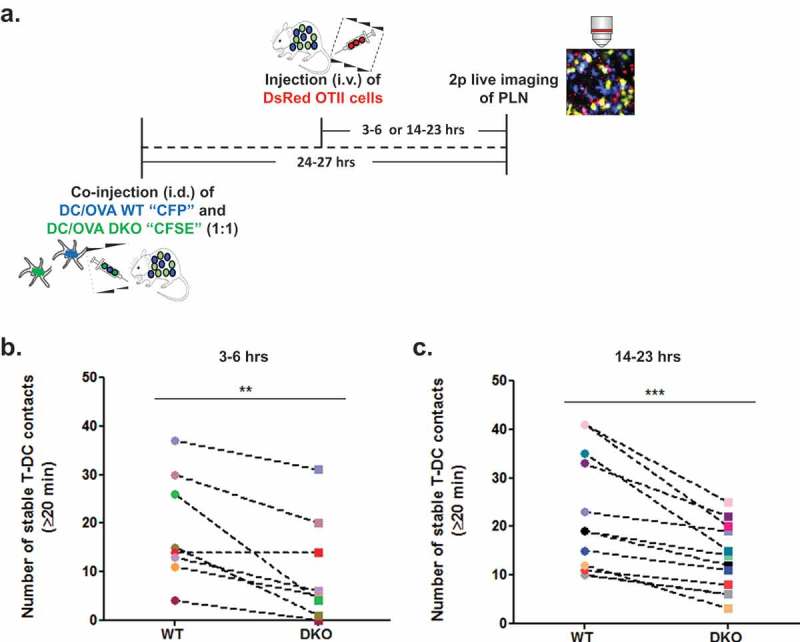
Stable OT-II conjugates with co-transferred LPS stimulated OVA peptide loaded WT and ICAM1/2 DKO DCs imaged at different time points post i.v. transfer of OT-II T cells. (a) A scheme depicting WT and ICAM 1/2 DKO migratory DCs loaded with a saturating dose of OVA peptide introduced intrafootpad 24–27 h prior to live imaging performed either 3–6 h or 14–23-h post i.v. injection of the OT-II T cells. (b) The number of stable conjugates depicted between WT or DKO migratory DCs and OT-II T cells injected either 3–6 h (left) or 14–23 h (right) prior to imaging. Stable conjugates were defined as T-DC pairs observed at the beginning of each imaging period and persisting for at least 20 min thereafter. Conjugates present in the same fields are shown in multiple experiments, each marked with a different symbol color. The number of T-DC conjugates in each field was normalized to identical WT and DKO DC density per field in each experiment. Left panel: ** p < 0.0088; Right panel: *** p < 0.0006. No conjugates were detected between polyclonal CD4 T cells and WT BM-DCs (not shown).

We next used intravital 2 photon imaging of popliteal lymph node T zones to analyze the total number of stable conjugates (i.e., lasting ≥ 20 min) of OT-II T cells with either co-transferred WT or ICAM-1/2 DKO BMDCs, each loaded with a saturating dose of the OT-II specific OVA peptide 323–339 [8] and injected 24–27 h earlier (Figure 2(a and b)). Notably, no conjugates were observed between polyclonal CD4 T cells and any of these DCs. Importantly, in spite of large variability between fields of view, significantly less conjugates (i.e., 46% fewer) formed between OT-II cells and OVA peptide loaded ICAM-1/2 DKO BMDCs than with OVA peptide loaded WT BMDCs present at the same fields of view (Figure 2(b) and Video 1). Fewer conjugates of OT-II and WT BMDCs were observed when these DCs were stimulated with LPS and loaded with low dose OVA peptide (Suppl. Figure 2). Similar numbers of conjugates, however, were generated by naïve OT-II cells with either WT or ICAM-1/2 DKO BMDCs loaded with this low dose OVA peptide (Suppl. Figure 2).

Given that OT-II T cells can establish partially ICAM-1 dependent conjugates with migratory BMDCs presenting high antigenic content, we next wished to compare both the number of these rare contacts and their dependence on DC-ICAMs at a later time point following adoptive transfer (i.e., 14–23 h after i.v. OT-II injection rather than our standard 3–6-h post i.v. injection). Notably, the numbers of stable conjugates by OT-II T cells and OVA peptide loaded BMDCs detectable at this late time point post i.v. injection were comparable to those observed 3–6 h after injection, suggesting that most of the stable OT-II-DC conjugates were reversible and therefore did not accumulate with time. Surprisingly, however, the dependence of the OT-II-DC conjugates on DC ICAMs did not vary with time (40% fewer OT-II-DC conjugates with ICAM deficient DCs than with WT DCs at this late time point vs. 46% fewer conjugates at the early time points (Figure 2(b and c)).Thus, naive OT-II cells that scanned these lymph nodes for OVA peptide loaded DCs for longer periods were not more prone to undergo in situ TCR mediated inside-out LFA-1 activation.

**Figure 3.**
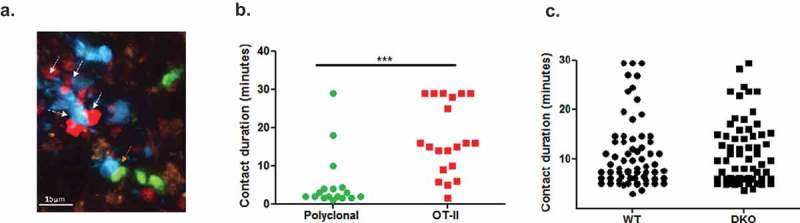
TCR stimulated arrests of OT-II on WT and ICAM KO BMDC inside lymph nodes analyzed by 2P microscopy. (a) A representative image from an intravital microscopy movie showing co-injected naïve DsRed OT-II T cells (white arrows) and non-specific polyclonal (GFP) CD4^+^ T cells (orange arrow) interacting with migratory WT LPS stimulated BMDCs (CFP) loaded with saturating OVA peptide in intact popliteal lymph nodes. (b) Contact durations of the different T cell subsets interacting with WT migratory DCs, as described in A, analyzed by intravital 2P microscopy (n = 2, representative of four movies). OVA-loaded WT BMDCS were introduced intrafootpad 24–27 h prior to live imaging performed 3–6-h post i.v. injection of the different T cells. (c) Contact durations of DS-Red OT-II CD4^+^ T cells with co-transferred LPS stimulated CFP WT and CFSE DKO BMDCs loaded with a saturating dose of an OT-II binding OVA peptide. OVA-loaded BMDCs were introduced intrafootpad 24–27 h prior to live imaging performed 3–6-h post i.v. injection of the OT-II T cells. The contact durations were determined by 2P imaging in six fields of view imaged in five mice (in three independent experiments).

We next used intravital 2 photon imaging to follow in greater detail the kinetics of contacts generated between the OT-II T cells and the various LPS-stimulated OVA peptide-loaded DCs injected 24 h earlier. As expected, migratory WT BMDCs could arrest OT-II T cells but not control co-injected polyclonal T cells when both groups of T cells entered these zones at comparable numbers (**Video 2**). Indeed, nearly all OT-II T cell contacts with migratory antigen loaded DC lasted longer than 5 min, whereas the polyclonal T cell-DC contacts, although frequent were shorter (Figure 3(a and b)). This result indicated that in our experimental setting all adhesive T-DC contacts lasting longer than 5 min are antigen specific . We next assessed the number and duration of contacts formed between similarly injected OT-II T cells and co-transferred OVA peptide loaded WT migratory DCs vs. ICAM-1/2 DKO BMDCs. Surprisingly, the ICAM-1 deficient migratory BMDCs stopped motile naïve OT-II CD4 T cells at comparable efficiencies as their WT DC counterparts (Figure 3(c) and Video 3) and the distribution of both short-lived and long-lived contacts between OT-II T cells and either WT or ICAM-1/2 DKO BMDCs was essentially indistinguishable (Figure 3(c)). Thus, the OT-II conjugates found to preferentially form with WT migratory DCs both at early and late time points following OT-II introduction into lymph nodes (Figure 2(b)) represent a subset of long-lived contacts, possibly LFA-1-ICAM-1 stabilized synapses lasting longer than 40 min, the upper limit of our real-time imaging of T cells and DCs inside lymph nodes.

**Figure 4.**
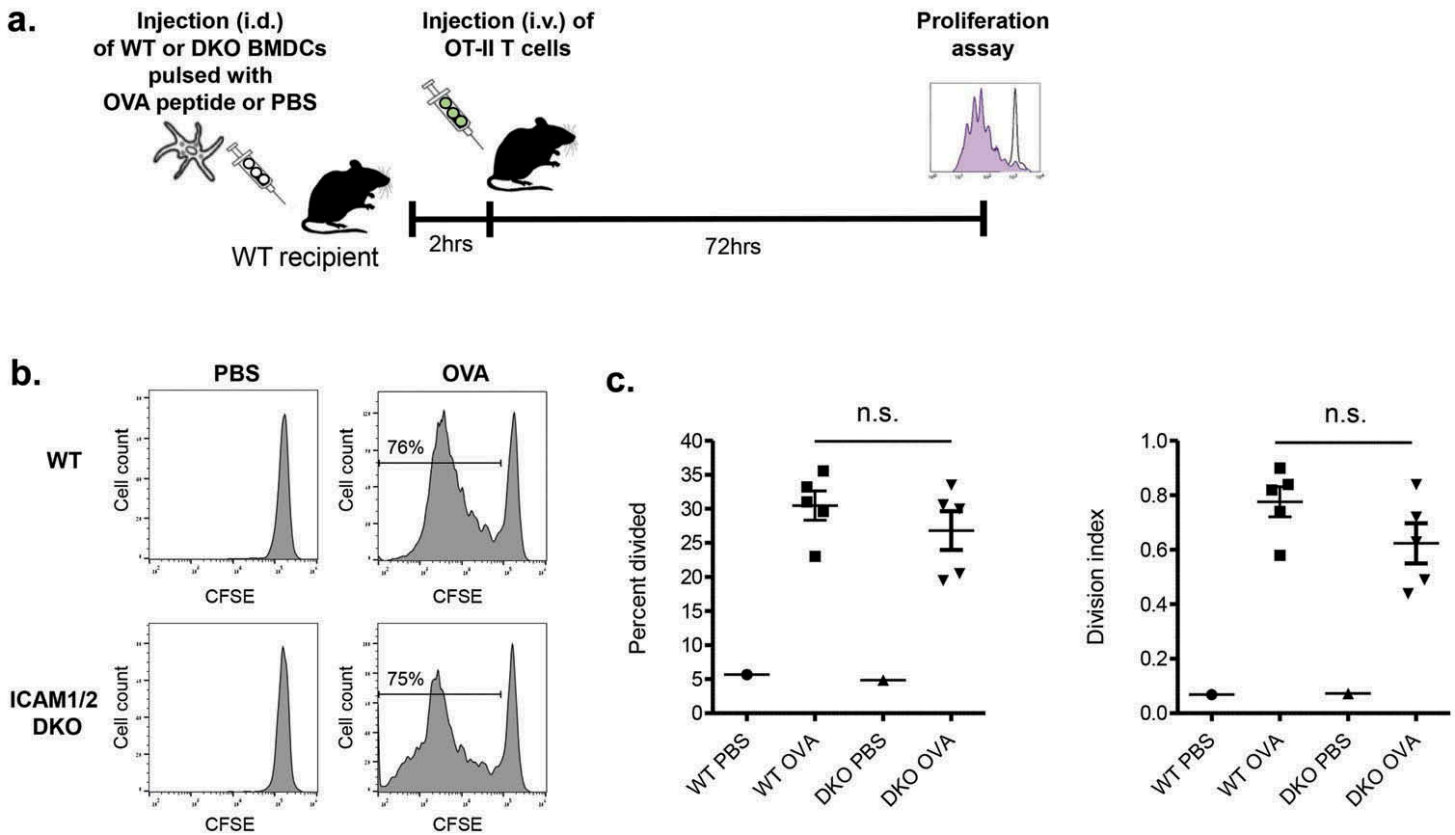
Proliferation of OT-II CD4 T cells inside lymph nodes transferred with LPS stimulated OVA peptide loaded skin migratory BMDCs (a) A scheme depicting the outline of the experiment. (b) Proliferation histograms of CFSE labeled OT-II CD4 T cells transferred 2 h after intrafootpad injection of WT BMDCs or DKO BMDCs each loaded with a saturating dose of OVA peptide. CSFE levels were determined 72 h after T cell injection. (c) The percentage of the OT-II CD4 T lymphocytes shown in (B) that divided at least once and their division index. n = 3. ns, nonsignificant by two-tailed test.

Finally, we wished to address if these rare ICAM-1 mediated OT-II-DC conjugates contributed to in vivo OT-II T cell proliferation inside these lymph nodes. Strikingly, similar division rates were observed for CFSE loaded OT-II cells 3 days after their entry into popliteal lymph nodes containing either migratory WT or ICAM-1/2 DKO BMDCs (LPS stimulated and loaded with high dose OVA peptide) (Figure 4(a-c)). These results collectively suggest that the presence of ICAM-1 or ICAM-2 on migratory BMDCs stimulated with a strong adjuvant (LPS) and presenting high density of antigenic moieties, is not obligatory for their ability to trigger in vivo proliferation of naïve cognate CD4 T cells engaging these DCs. Notably, during the 3 day period, the original OVA peptide loaded BMDCs could have transferred part of their cargo to neighboring WT lymph node DCs. Nevertheless, these DCs were unlikely to compete with the adoptively transferred WT and ICAM deficient BMDCs on global OT-II activation inside the lymph nodes. Furthermore, WT DCs loaded with low OVA peptide failed to generate any ICAM dependent contacts with OT-II cells (Suppl. Figure 2).

Taken together, our findings suggest that unlike resident adjuvant stimulated lymph node DCs [13], LPS stimulated migratory DCs with high levels of ICAM-1, MHC-II and loaded antigenic peptide can generate ICAM-1 mediated synapses with naïve OT-II, possibly by triggering the LFA-1 on these T cells to undergo proper inside-out activation [6]. The contribution of this key LFA-1 ligand for T cell-DC synapse formation appears to depend on the type and the stimulation state of the DC, the surface density of ICAM-1, as well as on the density of cognate pMHC complexes it presents. It is also possible that since LFA-1 adhesions are facilitated by venular shear forces [24–26], the low shear flow inside lymph nodes restricts LFA-1 inside-out activation in naïve CD4 T cells encountering antigen presenting DCs. Our results rule out, however, a role for ICAM-1 stabilized T-DC synapses as essential for naïve CD4 T cell priming and proliferation. These results are consistent with an old study that reported defective naïve CD8 contacts with ICAM-1 deficient BMDCs in spite of normal proliferation inside ICAM-1 deficient lymph nodes [11]. DC ICAM-1 may be critical, however, for subsequent memory acquisition by proliferating T cells [11] possibly because precursors of these memory T lymphocytes express more activated LFA-1 capable of engaging with DC ICAMs and non-hematopoietic stromal ICAM-1. Early on after entering lymph nodes, individual clones of naïve CD4 and CD8 T cells may avoid using their LFA-1 to generate high avidity LFA-1-ICAM interactions with resident and migratory DCs in order to keep high motility and effective scanning of the lymph node T zone for the rare DCs that present their cognate antigen [27]. How distinct effector CD4 and CD8 T cells and memory precursors of these subsets fine tune their LFA-1 hours to days after entry to lymph nodes and the outcomes of this fine-tuning is still an open issue [1] which could be useful for improved T cell vaccination.

## Materials and methods

### Mice

Mice (C57BL/6 background) were maintained in a pathogen-free facility and all animal procedures were approved by the Animal Research Committee at the Weizmann Institute of Science. The ICAM-1 and ICAM-2 double-deficient mice (ICAM-1/2-DKO mice) were generated as previously described [13] and were back crossed for 10 generations. DsRed OT-II mice were prepared as previously described [13]. GFP and CFP transgenic mice were obtained from Jackson Laboratories.

### BM cultures, LPS stimulation and t cell isolation

BMDCs were extracted from the tibia and fibula bones of euthanized WT CFP and ICAM-1/2-DKO mice, seeded in non-tissue culture treated bacteriological Falcon plates (Corning, Cat. 351029) and cultured in DC medium (RPMI 1640 medium, Cat. 01–100-1A) with 10% FCS (Cat. 04–001-1A), β-mercaptoethanol (50uM, Sigma-Aldrich, Cat. M3148), L-glutamine (2 mM, Cat. 03–020-1B), Penicillin G Sodium Salt (10,000 IU/ml), Streptomycin Sulfate (10 mg/ml) and amphotericin B (25 μg/ml) (PSA B solution, Cat. 03–033-1B) were all from Biological Industries. The DC culture was supplemented with recombinant mouse GM-CSF (20 ng/ml) and IL-4 (20 ng/ml) (Peprotech, Cat. 214–14-20 and 315–03-05, respectively). On day 9 BMDCs were transferred to tissue culture 6 well plates (Cellstar, Cat. 657–160) and stimulated with LPS (0.5ug/ml) (Sigma-Aldrich, strain 0111:B4, Cat. L2630-100MG). Both polyclonal WT GFP T cells and CD4+ OT-II DsRed T cells were purified from spleens of respective 24–48-week old donor mice with MACS CD4^+^ T cell isolation kit (Miltenyi Biotec, Cat. 130–104-454).

### Intravital two-photon microscopy of lymph nodes

WT and ICAM 1/2 DKO BMDCs (pre-stimulated with LPS for 24 h) were loaded for 45 min with saturating levels of the OT-II specific chicken OVA 323–339 peptide (1uM in 1ml containing 10^6^ DCs) (Sigma-Aldrich, Cat. O1641-5MG). All DCs were CD11c+ and MHC-II+. 2x10^6^ of DKO BMDCs were labeled with CFSE (5 μM) (Molecular Probes, Cat. 11524217) as described [28] and co-injected intradermally into the footpads of 10–12-week old WT mice with 2 × 10^6^ WT CFP DCs. OT-II DsRed T cells (2x10^6^) were i.v. injected into the same recipient mice 3–6 or 14–23-h prior to intravital imaging (24–27-h post BMDC intrafootpad injection). Popliteal LNs were surgically exposed, immobilized, covered with a cover slip and placed under a Zeiss LSM 880 upright microscope using a 20 × 1.05 NA plan objective. Live imaging of the popliteal lymph node was performed as previously described [13] and was conducted for 40 min to minimize phototoxic side effects. Images were acquired once per min as 50–100 µm Z-stacks with 5 µm steps between each Z-plane. Our quantitation protocol required that DCs be continuously trackable in the imaging field. Durations of contacts between OT-II T cells and individual DCs were determined manually using Imaris software (Bitplane). An adhesive contact was defined as a T cell-DC cell interaction lasting longer than 3 min. Stable conjugates were defined as T-DC pairs observed in the beginning of each imaging period and persisting for at least 20 min thereafter.

### In vivo proliferation assay

10^6^ LPS stimulated WT or DKO BMDCs were loaded with OVA peptide (1uM in 1 ml containing 10^6^ DCs) and intra-footpad injected into C57BL/6 WT mice. 10^7^ spleen derived OT-II T cells were labeled with CFSE and injected i.v. into C57BL/6 WT mice 2-h post BMDC transfer. 72 h later the CFSE labeled OT-II cells were recovered from total popliteal lymph node cell suspensions and analyzed on a CytoFLEX Cytometer (Beckman Coulter). Post-acquisition analysis was performed using FlowJo software (Tree Star, Inc.). ‘Percent Divided’ refers to the percentage of the original T cell population that underwent at least one division. ‘Division Index’ is defined as the average number of cell divisions that a cell in the original population has undergone including non-dividing T cells.

### Statistical analysis

Data were statistically analyzed using GraphPad Prism software and using a two-tailed Student’s t test. Significance was set to p < 0.05.
