# Supplementary material for: Stable contacts of naïve CD4 T cells with migratory dendritic cells are ICAM-1-dependent but dispensable for proliferation in vivo
Source: Cell Adh Migr. 2019 Jul 31;13(1):315–21. doi: 10.1080/19336918.2019.1644857 (PMC6682365; doi:10.1080/19336918.2019.1644857)
Supplement: Supplemental Material [file kcam-13-01-1644857-s002.zip › Supplementary files/Supplementary Figure 1_190506.pptx]

## Slide 1
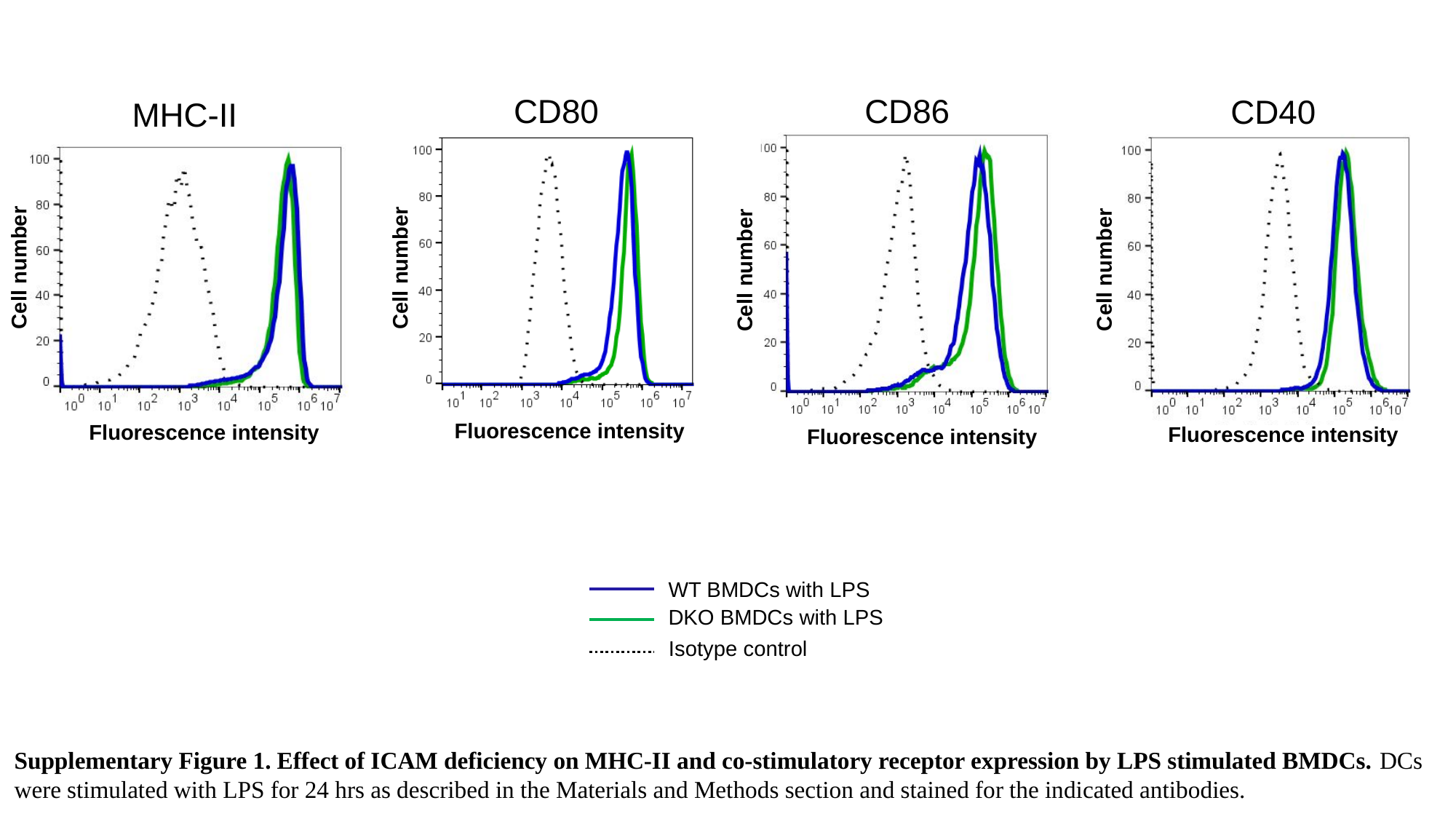

CD80
CD86
CD40
MHC-II
Cell number
Cell number
Cell number
Cell number
Fluorescence intensity
Fluorescence intensity
Fluorescence intensity
Fluorescence intensity
WT BMDCs with LPS
DKO BMDCs with LPS
Isotype control
Supplementary Figure 1. Effect of ICAM deficiency on MHC-II and co-stimulatory receptor expression by LPS stimulated BMDCs. DCs
were stimulated with LPS for 24 hrs as described in the Materials and Methods section and stained for the indicated antibodies.
