# Supplementary material for: Stable contacts of naïve CD4 T cells with migratory dendritic cells are ICAM-1-dependent but dispensable for proliferation in vivo
Source: Cell Adh Migr. 2019 Jul 31;13(1):315–21. doi: 10.1080/19336918.2019.1644857 (PMC6682365; doi:10.1080/19336918.2019.1644857)
Supplement: Supplemental Material [file kcam-13-01-1644857-s002.zip › Supplementary files/Supplementary Figure 2_190506.pptx]

## Slide 1
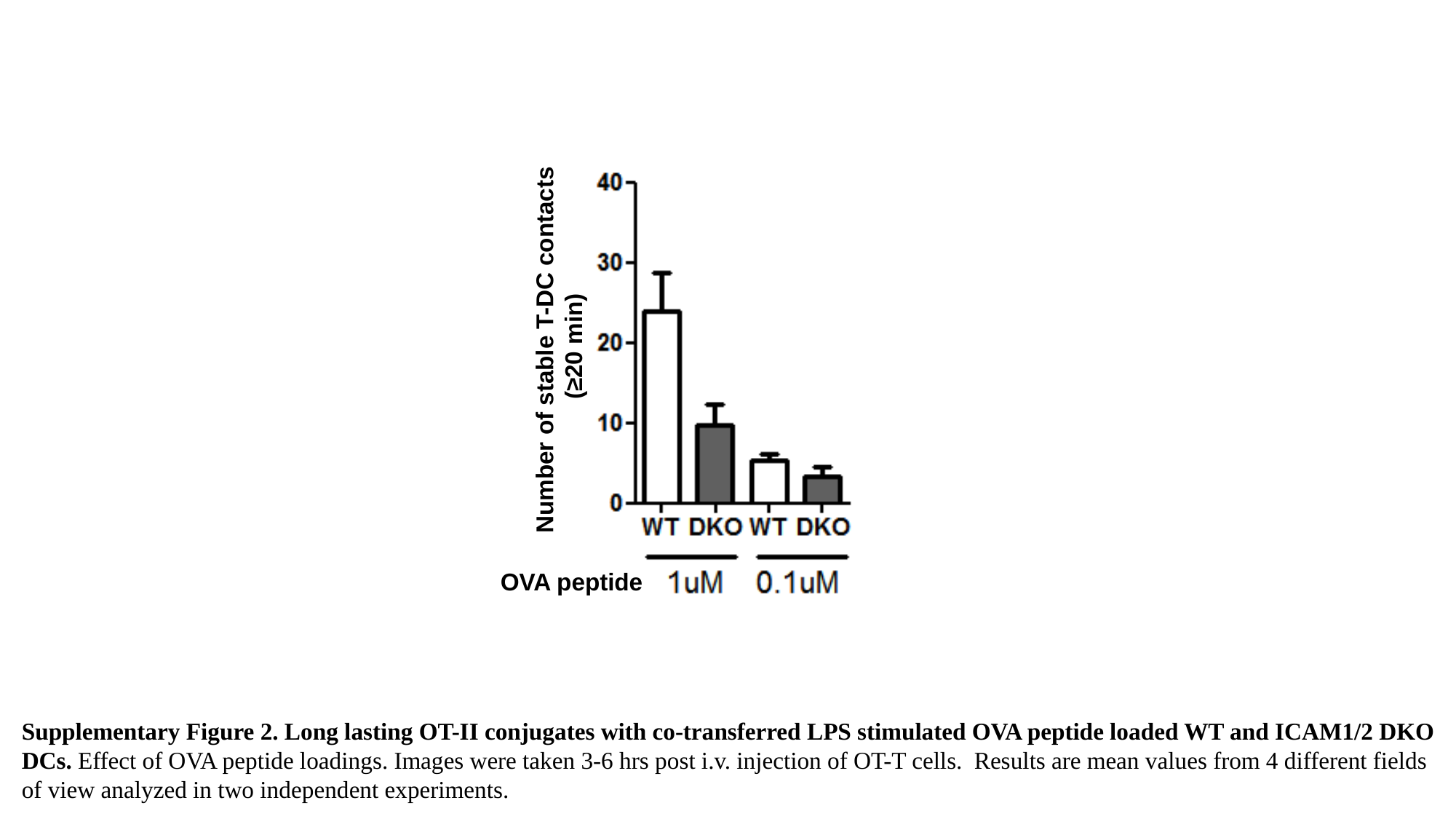

Number of stable T-DC contacts
(≥20 min)
OVA peptide
Supplementary Figure 2. Long lasting OT-II conjugates with co-transferred LPS stimulated OVA peptide loaded WT and ICAM1/2 DKO DCs. Effect of OVA peptide loadings. Images were taken 3-6 hrs post i.v. injection of OT-T cells. Results are mean values from 4 different fields of view analyzed in two independent experiments.
