# Supplementary material for: Stable contacts of naïve CD4 T cells with migratory dendritic cells are ICAM-1-dependent but dispensable for proliferation in vivo
Source: Cell Adh Migr. 2019 Jul 31;13(1):315–21. doi: 10.1080/19336918.2019.1644857 (PMC6682365; doi:10.1080/19336918.2019.1644857)
Supplement: Supplemental Material [file kcam-13-01-1644857-s002.zip › Supplementary files/Supplementary Materials_190506.docx]

**Supplementary Information**

**Supplementary Methods**

**Flow Cytometry and antibodies**

The following fluorescently-conjugated anti-mouse primary antibodies were purchased from Biolegend: CD45 (Clone 30-F11, Cat. 103134), I-Ab (clone AF6-120.1, Cat. 116416), CD80 (clone 16-10A1, Cat. 104714), CD86 (clone GL-1, Cat. 105028), CD40 (clone 3/23, Cat. 124622), CD11c (clone N418, Cat. 117310), CD54 (ICAM-1, clone YN1/1.7.4, Cat. 116108), CD102 (ICAM-2, clone 3C4, Cat. 105609), CD19 (clone 6D5, Cat. 115520), CD11b (clone M1/70, Cat. 101208), CD40 (clone3/23, Cat. 124622), CD4 (clone RM4-5, Cat. 100526), CD8a (clone 53-6.7, Cat. 100723), CD103 (clone 2E7, Cat. 121406), and isotype controls: IgG2b,κ (clone [MRG2b-85](https://www.biolegend.com/en-us/search-results?Clone=MRG2b-85), Cat. 408214) and IgG2a,κ (clone [RTK2758](https://www.biolegend.com/en-us/search-results?Clone=RTK2758), Cat. 400506, 407112). Unconjugated CD40 mAb (Bio X cell, clone FGK4.5/FGK45, Cat. BE0016-2) was intrafootpad injected to activate DCs in vivo as previously described [13]. For analysis of surface expression of various proteins and ex vivo proliferation assay, cells were labeled with fluorescent primary antibodies (10μg/ml), washed, resuspended in fluorescence-activated cell sorting (FACS) buffer (PBS-/-, 1% BSA, 5mM EDTA, and 0.01% sodium azide), and analyzed on the CytoFLEX S Flow Cytometer (Beckman Coulter). Data was acquired with CytExpert software (Beckman Coulter) and post-acquisition analysis was performed using FlowJo software (Tree Star,Inc.).

**Supplementary Video Legends**

**Video 1.** Representative movie of dsRed OT-II (red) stably conjugated to either LPS stimulated CFP WT BMDCs (marked by white circles) or to CFSE ICAM DKO BMDCs (marked by orange circles). Both types of DCs were loaded with saturating doses of OVA peptide and co-injected into the footpad 24 hrs before intravital imaging. The movie corresponds to Fig. 2B. Bar, 50 μm.

**Video 2.** Representative movie of dsRed OT-II (red) and GFP-polyclonal (green) CD4^+^ T cells migrating inside the T zone of the popliteal lymph node into which LPS stimulated CFP BMDCs saturated with OVA peptide were injected into the footpad 24 hrs before intravital imaging as described in the Materials and Methods section and with results depicted in Fig. 3B. White arrows denote stable OT-II arrests on representative DCs. Bar, 30 μm.

**Video 3.** Representative movie of dsRed OT-II (red) T cells migrating inside the T zone of the popliteal lymph node into which LPS stimulated CFP WT BMDCs and CFSE ICAM DKO BMDCs each saturated with OVA peptide were co-injected into the footpad 24 hrs before intravital imaging as described in the Materials and Methods section and with results depicted in Fig. 3C. White and orange arrows denote stable OT-II arrests on WT and ICAM DKO DCs, respectively. Bar, 20 μm.
